# The Role of Lactoferrin in Combating *Candida* spp. Infections Through Regulation of Oxidative Stress, Immune Response, and Nutritional Support in Women and Newborns

**DOI:** 10.3390/molecules30112416

**Published:** 2025-05-31

**Authors:** Anna Długosz, Joanna Wróblewska, Paweł Kołaczyk, Weronika Wróblewska

**Affiliations:** 1 Department of Food Industry Technology and Engineering, Faculty of Chemical Technology and Engineering, Bydgoszcz University of Science and Technology, 3 Seminaryjna St., 85-326 Bydgoszcz, Poland; pawel.kolaczyk@pbs.edu.pl; 2 Department of Medical Biology and Biochemistry, Faculty of Medicine, Ludwik Rydygier Collegium Medicum in Bydgoszcz, Nicolaus Copernicus University in Toruń, 24 Karłowicza St., 85-092 Bydgoszcz, Poland; 3 Student Research Club of Medical Biology and Biochemistry, Department of Medical Biology and Biochemistry, Faculty of Medicine, Ludwik Rydygier Collegium Medicum in Bydgoszcz, Nicolaus Copernicus University in Toruń, 24 Karłowicza St., 85-092 Bydgoszcz, Poland; 316714@stud.umk.pl

**Keywords:** antifungal activity, bioactive peptides, *Candida* spp., fungal infections, lactoferrin, mucosal immunity, neonates, pregnant women

## Abstract

Lactoferrin (LF) is a natural glycoprotein with strong antimicrobial, immunomodulatory, and nutritional potential and is widely present in milk and mucosal secretions. This paper aims to review the current knowledge on the application of lactoferrin and its bioactive peptides in the context of fungal infections caused by *Candida* spp., focusing on newborns and pregnant women as high-risk groups. The multifaceted mechanisms of LF action are discussed, including iron chelation, destabilization of fungal cell membranes, and modulation of the immune response. Additionally, data demonstrating the effectiveness of LF in the prevention and supportive treatment of *Candida* spp. infections are presented.

## 1. Introduction

Fungal infections caused by yeasts of the genus *Candida*, particularly *Candida albicans*, represent a significant health concern in both women and newborns [1,2]. While *C. albicans* remains the most common etiological agent of these infections, an increasing prevalence of non-*albicans* species such as *Candida glabrata*, *Candida krusei*, *Candida parapsilosis*, and *Candida tropicalis* is being observed, especially in patients with recurrent infections or poor response to antifungal therapy. These species are often more resistant to azole treatments and tend to present with milder clinical symptoms [3].

The yeast form of *Candida* spp. is a natural component of the lower female genital tract. However, under favorable conditions such as disruption of the microbiota, elevated estrogen levels, or increased vaginal pH, it may convert into the hyphal form and cause symptomatic infection [3,4]. The most common clinical manifestation of fungal infection in women is vulvovaginal candidiasis. It is estimated that up to 75% of women experience it at least once in their lifetime, and in 5 to 10% of cases, it becomes recurrent [1]. Pregnancy is a period of exceptionally high risk for such infections. Hormonal and immunological changes associated with pregnancy facilitate vaginal colonization by *Candida* yeasts [3,5]. Lower genital tract infections during pregnancy are linked to unfavorable outcomes such as miscarriage, premature rupture of membranes, and preterm birth [6]. Vaginal candidiasis is most frequently diagnosed in the first trimester of pregnancy and much less commonly in the third trimester, which may suggest increased susceptibility to fungal colonization in early pregnancy [7]. However, this finding is based on cross-sectional data, and it remains unclear whether the observed decrease later in pregnancy reflects actual clearance, reduced detection, or underdiagnosis. Information on how *Candida* spp. colonization changes over time during pregnancy is limited. One study that sampled women at multiple time points found relatively stable colonization rates throughout pregnancy [8]. Infections caused by *Candida* spp. can also be asymptomatic, meaning many cases remain undiagnosed [7]. The defense of vaginal tissues against *Candida* spp. invasion involves the cooperation of innate and adaptive immune mechanisms. These include the production of cytokines (such as interleukin 17 (IL-17) and interleukin 23 (IL-23)), the presence of antibodies, the activity of antimicrobial peptides (AMP), and the presence of probiotic bacteria from the *Lactobacillus* genus [4].

Although direct invasion of placental tissues by yeasts has not been confirmed, research indicates that *Candida* spp. vaginal infection during pregnancy may disrupt proper placental development, leading to pregnancy complications and neonatal colonization [6,7]. Colonization of the gastrointestinal tract by *Candida* spp., especially in extremely premature infants, is a significant risk factor for the development of invasive candidiasis [9]. Epidemiological data confirm that early-onset neonatal invasive candidiasis, occurring within the first week of life, primarily affects infants with very low birth weight (<1000 g) and is often caused by *C. albicans* infection [10]. Mortality associated with neonatal invasive candidiasis can reach up to 75% [2]. *C. albicans* naturally colonizes healthy individuals’ gastrointestinal tract, mucous membranes, and skin. However, disturbances in immune or microenvironmental balance can lead to a transition of *Candida* spp. from a commensal to a pathogenic form, resulting in superficial infections and life-threatening systemic candidiasis [11]. According to studies, *Candida* spp. is present in the oral cavities of infants and newborns and shows an increased tendency to overgrow in children with immune or microbiota imbalances [11]. One of the most common superficial candidiasis manifestations in infants is oral thrush. The lesions affect the mucous membranes of the throat, gums, cheeks, lips, tongue, and lip corners and appear as white plaques resembling curdled milk [12]. Data from the literature report that the prevalence of pseudomembranous candidiasis ranges from 4% to 15% [13]. Pathogen transmission within the mother–infant–mother loop may occur in breastfeeding women, whereby *C. albicans* detected in the infant’s oral cavity can be transmitted to the mother’s nipples [14].

In recent years, increasing attention has been paid to lactoferrin (LF), a natural antimicrobial protein found in milk, mucus, and other bodily secretions. LF is attributed with numerous functions, including microbistatic and microbicidal activity against a range of pathogens, modulation of the immune response (through both anti-inflammatory and pro-inflammatory mechanisms), support for hematopoiesis, wound healing, bone metabolism, and potential anticancer and antioxidant effects [15]. In newborns, especially those who are breastfed, lactoferrin in maternal milk is an antimicrobial agent. It provides nutritional and immunological support that protects against colonization by pathogenic yeasts. LF exhibits broad antifungal activity against several *Candida* species, including *C. albicans*, *C. glabrata*, and *C. tropicalis* [16].

This paper aims to discuss the role of LF in proper nutrition and the prevention supportive treatment of infections caused by *Candida* spp., both in women of reproductive age and newborns. In the context of increasing reports of yeast resistance to conventional antifungal therapy, the use of lactoferrin (both human lactoferrin (hLf) and bovine lactoferrin (bLf)) may represent a promising strategy to support treatment and reduce the recurrence of infections within the urogenital and gastrointestinal tracts in both pregnant women and infants.

## 2. Biological Activity of Lactoferrin and Its Mechanisms of Action in the Human Body

LF is a multifunctional, non-heme iron-binding glycoprotein belonging to the transferrin family and is considered an AMP [17,18]. One of its most important functions is its involvement in iron metabolism [15]. LF exists in an iron-saturated form (holo-LF) and an iron-free form (apo-LF). This protein binds iron with high affinity in a reversible manner, stabilizing the molecule and making it less susceptible to enzymatic degradation and thermal denaturation. Unlike transferrin, LF retains iron even in acidic environments such as the intestine and supports transport through a specific receptor in enterocytes. After endocytosis, iron is released by reducing Fe^3+^ to Fe^2+^ [18]. Notably, Fe^2+^ is a more reactive form that can generate reactive oxygen species (ROS). Therefore, the iron-chelating activity of LF reduces ROS formation by maintaining iron in a less reactive state, thereby preventing oxidative stress. As such, LF plays an essential role in iron metabolism and in protecting cells from oxidative damage [18]. By binding free iron, LF limits its participation in the Fenton reaction, leading to the formation of highly harmful hydroxyl radicals. LF binds iron stably in neutral pH environments typical of most tissues, preventing its involvement in redox reactions. Through this ability, LF protects cells from iron-induced damage and supports the maintenance of cellular function, particularly under conditions of disturbed iron homeostasis [18].

LF releases previously bound iron in the acidic environment of phagolysosomes (e.g., in neutrophils and macrophages), where the pH drops to 3–4. The resulting Fe^2+^ ions can generate ROS, which possess vigorous cytotoxic activity against pathogens. Thus, beyond its protective role for host cells, LF enhances the microbicidal potential of phagocytes, supporting the immune response [15]. Additionally, LF acts as a potent antioxidant not only by directly scavenging ROS but also by enhancing the activity of endogenous antioxidant enzymes such as superoxide dismutase (SOD) and glutathione peroxidase. These effects reduce lipid peroxidation products and hydrogen peroxide concentrations in the blood. A general increase in plasma antioxidant capacity has also been observed, underscoring LF’s systemic protective role and its potential in preventing chronic diseases associated with oxidative stress [18].

LF is synthesized and secreted by both neutrophils and glandular epithelial cells. Its presence has been demonstrated in the nasal mucosa, bronchi, skin (particularly in sweat glands), and various other bodily secretions, highlighting its essential role in local and mucosal immunity [19,20,21]. In women with a regular menstrual cycle, lactoferrin levels in cervicovaginal secretions fluctuate according to estrogen concentration, increasing during the proliferative (follicular) phase and decreasing during the secretory (luteal) phase. Additionally, lactoferrin is present in the amniotic fluid and the thick cervical mucus plug during pregnancy, suggesting its involvement in protecting the early fetal environment [22]. Table 1 presents lactoferrin concentrations in selected human body fluids.

LF released from neutrophil granules enters the bloodstream and sites of inflammation, which actively contributes to regulating immune responses [18,23]. In a healthy individual, approximately 5 g of neutrophil-derived LF is produced daily, with about 0.5 g released during neutrophil degranulation [15]. LF acts on various immune cells by binding to their surface receptors and modulating intracellular signaling pathways, particularly those involving Toll-like receptors (TLRs), which can either activate or suppress inflammatory responses depending on the immunological context [24]. In macrophages, it engages both TLR4-related and alternative signaling routes. For example, the expression of the CD40 receptor depends on TLR4, whereas the production of IL-6 does not, indicating distinct mechanisms of action [25]. LF also stimulates NK cells, enhancing their cytotoxic function and aiding in the elimination of infected cells [5]. It primarily supports innate immunity by targeting key effector cells such as neutrophils, macrophages, NK cells, and dendritic cells, which are essential for initiating adaptive responses [23,25] Moreover, LF functions as an alarmin by signaling danger and promoting the recruitment and activation of antigen-presenting cells like dendritic cells, thereby indirectly enhancing T-cell activation and the development of adaptive immunity [5,26].

Due to the increased demand for iron during pregnancy, its deficiency may lead to anemia and perinatal complications. Therefore, LF is considered a safe and effective alternative to traditional iron supplementation and may help avoid the need for higher iron doses during pregnancy. It supports iron absorption and regulation, improves hematological parameters, and is better tolerated than conventional iron preparations [5]. Combined oral and intravaginal administration of LF may serve a preventive function in preterm birth, partly by lowering interleukin-6 (IL-6) and prostaglandin levels in cervicovaginal secretions [5,27].

LF, as an antimicrobial protein, influences the composition of the genital tract microbiota through both direct and indirect prebiotic activity. It directly promotes the growth of probiotic strains such as *Lactobacillus* spp. and *Bifidobacterium* spp. and indirectly limits the growth of pathogenic microorganisms. Importantly, it acts selectively by eliminating harmful microbes without disturbing the balance of the natural probiotic microbiota [5,22].

**Table 1:** Lactoferrin concentration in selected human body fluids.

Body Fluid		LF Concentration	Ref.
Vaginal mucus	Before menstruation	3.8–11.4 µg/mg	[17]
	After menstruation	62.9–218 µg/mg	[17]
	During contraception use	19.8 µg/mg	[17]
Amniotic fluid	Early pregnancy	1–2 μg/ml	[22]
	From the 32nd week of pregnancy until delivery	5–15 μg/ml	[22]
Cervical mucus plug		10–1000 µg/ml	[22]
Human colostrum		6–7 g/L	[5,28]
Mature human milk		1–3 g/L	[5,28]
Saliva		10–100 µg/mL	[22]
Blood		10^−3^–200 µg/mL	[17]

Lactoferrin (LF).

## 3. The Importance of Lactoferrin in Nutrition

Human milk is widely regarded as the most optimal form of infant nutrition, supporting their healthy development [29]. It contains varying concentrations of LF [28]. This protein may be higher in the milk of mothers who deliver preterm than those who give birth at term [28]. The protective potential of lactoferrin may be maintained or even enhanced during later stages of breastfeeding through maternal milk delivery [30]. Breastfed infants have a lower incidence of infections, necrotizing enterocolitis (NEC), and sudden infant death syndrome, which contributes to reduced neonatal mortality. In later life, breastfeeding is associated with a significantly lower risk of developing various chronic diseases, such as overweight and obesity, type I and II diabetes, asthma (which is hypothesized to be reduced, particularly in early childhood), lymphoma, Hodgkin’s disease, acute lymphoblastic leukemia, and elevated cholesterol levels [31,32,33]. Breastfeeding provides benefits not only for the infant but also for the mother. Shortly after delivery, it helps shorten the duration of postpartum bleeding, accelerates uterine involution, supports lactational amenorrhea as a natural form of contraception, facilitates a return to pre-pregnancy body weight, and may alleviate symptoms of postpartum depression [31,34,35,36,37]. Moreover, studies indicate that lactation has long-term positive effects on maternal health. It is believed to reduce the risk of breast and ovarian cancers and osteoporosis and also lower the incidence of hip fractures, hypertension, diabetes, and lipid disorders [31,38,39,40]. Both breastfeeding and formula feeding have their advantages and limitations. The infant-feeding method should result from an informed decision made by the parents in consultation with healthcare professionals, considering the child’s health, family lifestyle, cultural context, and available resources [41]. It is also worth noting that after breastfeeding ends, the availability of lactoferrin in the daily diet decreases sharply. Cow’s milk contains only 0.1–0.3 mg/mL of LF, and thermally processed products such as UHT milk are almost entirely devoid of this protein due to its denaturation and loss of iron-binding capacity [5,15]. Pasteurization of milk at 62.5 °C for 30 min can reduce LF levels by up to 88%, significantly diminishing its anti-infective and antioxidant potential [42]. In response to the low lactoferrin content in cow’s milk, infant formula manufacturers enrich their products with bLf and recombinant human lactoferrin (rhLf) [43]. Since bLf shares 70–74% amino acid sequence identity with hLf, it is widely used as an ingredient in infant formulas and food products for special medical purposes [23,28,44]. Although the level of bLf in commercially available infant formulas is significantly lower than in natural human milk [28], clinical studies have shown that bLf supplementation can improve hematological parameters, support immune system maturation, and reduce the risk of infections and sepsis in preterm infants [45,46]. King et al. [45] investigated the effects of infant formula enriched with LF on healthy infants born at 34 weeks of gestation and enrolled in a pediatric clinic at 4 weeks of age. The infants were randomly assigned to one of two groups: the intervention group, consisting of 26 infants, received formula containing 850 mg/L of LF, while the control group, also consisting of 26 infants, received standard cow’s milk-based formula containing 102 mg/L of LF. Supplementation was administered over a 12-month period. Infants fed with LF-enriched formula showed a significantly higher hematocrit level at 9 months of age, along with a trend toward higher hemoglobin levels. In a prospective, randomized trial, Akin et al. [46] demonstrated that daily supplementation with bLf (200 mg/day) in very-low-birth-weight preterm infants significantly reduced the incidence of hospital-acquired sepsis and led to an increase in the level of T-regulatory cells, which are essential for immune balance and intestinal protection against inflammation. Numerous studies have confirmed that bLf supplementation in preterm infants decreases the incidence of late-onset sepsis and NEC, improves survival rates, and does not cause adverse effects [47]. A systematic review and meta-analysis published in 2018 identified nine randomized controlled trials involving a total of 1834 preterm infants. The pooled analysis demonstrated that bLf supplementation significantly reduced the incidence of NEC and late-onset sepsis (LOS) and lowered the risk of hospital-acquired infection and infection-related mortality in preterm infants without evident adverse effects [48]. It is worth noting, however, that more recent studies have yielded mixed results. A study published in 2019—a large, multicenter, double-blind, randomized controlled trial (the ELFIN trial)—found no significant effect of bLf supplementation on the incidence of late-onset infections or other complications, such as NEC or mortality, in very preterm infants. The authors concluded that these findings do not support the routine use of bLf in this population [49]. Similarly, a 2022 meta-analysis reported that enteral lactoferrin supplementation was associated with a significant reduction in LOS but did not significantly affect the incidence of NEC or all-cause mortality [50]. These discrepancies suggest that while earlier studies demonstrated promising results regarding bLf supplementation, more recent research presents a more nuanced picture. Factors such as study design, population differences, and variations in supplementation protocols may contribute to these differing outcomes. Therefore, ongoing research and updated meta-analyses are essential to fully understand the efficacy and safety of bLf supplementation in preterm infants. Infants fed formulas enriched with lactoferrin experience fewer respiratory tract infections than those receiving standard formula milk [43]. However, the optimal dose of bLf for effective prevention and therapeutic support has not yet been established [28]. In a study conducted by Gao et al. [51], it was demonstrated that human milk supplemented with a bovine colostrum-based fortifier (bCF) exhibited more potent antimicrobial activity against pathogens commonly associated with neonatal sepsis, such as *Escherichia coli*, *Staphylococcus epidermidis*, and *Enterococcus faecalis*. Although this study did not assess the antifungal effects of lactoferrin, the observed outcomes suggest the general immunoprotective potential of bovine colostrum. Notably, the antimicrobial effect persisted even after bCF was supplemented with iron, suggesting that lactoferrin’s activity is not solely dependent on iron chelation but also involves synergy with other bioactive components of colostrum, such as osteopontin and growth factors. The authors suggest that such enrichment of milk may modulate gut microbiota composition and enhance the intestinal barrier, thereby reducing the risk of systemic infections and NEC in preterm infants [51].

The U.S. Food and Drug Administration and the European Food Safety Authority have recognized lactoferrin as safe for use as a food additive and dietary supplement. Daily lactoferrin intake in doses ranging from 100 mg to 4.5 g has shown no signs of toxicity [5]. According to the literature, hLf can be administered orally and parenterally (intravenously, subcutaneously, topically to the skin or wounds, and for body cavity rinses). In contrast, bLf, due to its non-human origin, is administered only orally or enterally [5].

In response to the limited availability of hLf after the breastfeeding period ends, the market for dietary supplements and food products enriched with this protein is expanding. Globally, products such as infant formulas, juices, yogurts, mineral waters, and even chewing gums with added lactoferrin are available, along with hygiene products and dermocosmetics [15].

LF present in fermented dairy products is one of the key dietary components supporting the development of probiotic microbiota. In addition to probiotic bacteria, these products provide prebiotics such as lactose, oligosaccharides, α-lactalbumin, lactoperoxidase, and lysozyme [5]. Lactoferrin exhibits prebiotic activity toward the intestinal microbiota and indirectly supports the beneficial composition of the vaginal microbiota. Oral administration of lactoferrin may further modulate immune responses within the gastrointestinal and reproductive tracts, which can offer long-term benefits in preventing recurrent infections of the urogenital tract [22]. Although lactoferrin may undergo partial enzymatic degradation in the adult gastrointestinal tract, studies indicate that its activity is locally preserved by generating immunoregulatory bioactive peptides [22]. These peptides, such as lactoferricin and lactoferrampin, retain or even enhance the antimicrobial activity of lactoferrin despite lacking iron-binding capacity, and they are resistant to further enzymatic degradation. Lactoferricin is naturally generated from LF during gastric digestion following oral administration [52].

**Figure 1:**
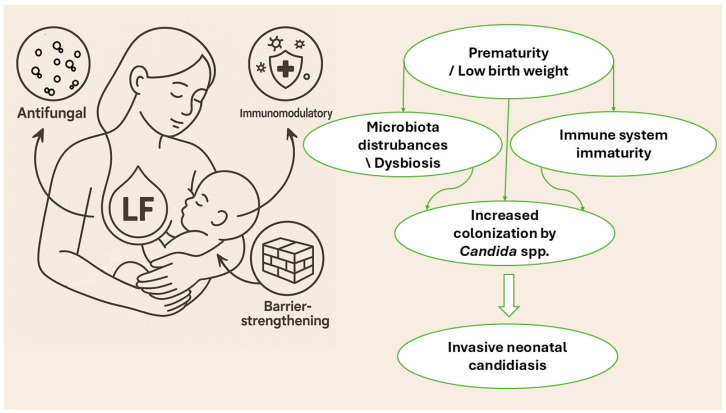
Risk factors for *Candida* spp. infection in newborns.

A characteristic feature of the neonatal period is the immaturity of the gastrointestinal system. This is manifested by higher gastric pH, reduced digestive enzyme activity, and increased gut–blood barrier permeability [15,43]. Additionally, in breastfed newborns, protein digestion is further limited due to the presence of protease inhibitors in colostrum. As a result, a significant portion of lactoferrin ingested with milk remains in the intestine and may enter the bloodstream [15]. LF, especially in its iron-saturated form, shows high resistance to proteolytic enzymes in the stomach, pancreas, and small intestine [15]. Studies have demonstrated that both hLf and bLf can be detected in the feces of infants in intact or partially digested form, confirming their stability in the neonatal gastrointestinal tract and potential for systemic activity [52]. Figure 1 shows the mechanisms of action of LF and the risk factors leading to invasive neonatal candidiasis.

## 4. Antifungal Functions of Lactoferrin: From Molecular Mechanisms to the Modulation of the Host Immune Response

LF exhibits antifungal properties whose mechanism, similar to its antibacterial action, is associated with the protein’s positively charged N-terminal polypeptide chain [53]. This region, particularly rich in arginine and tryptophan residues, interacts electrostatically with negatively charged components of the fungal membrane and cell wall (e.g., glucans or mannans), leading to lipid structure destabilization, increased permeability, cytoplasmic leakage, and, ultimately, cell death [17,54]. Additionally, lactoferrin can induce fungal cell apoptosis through chromatin condensation, DNA fragmentation, and the accumulation of ROS [55].

Biofilm formation is one of the primary virulence factors of microorganisms and plays a significant role in the persistence and treatment resistance of infections. In *C. albicans*, this process begins with an early phase during which yeast cells adhere to a surface and form initial microcolonies [56]. The synthetic peptide hLF1-11, derived from the N-terminal domain of human LF, has been shown to interfere with this phase by inhibiting cell adhesion and disrupting the initial steps of biofilm development. During this early stage, hLF1-11 effectively suppresses biofilm formation in a dose-dependent manner at concentrations ranging from 44 to 88 mg/L [57]. As the biofilm develops into the intermediate phase, yeast cells begin producing extracellular polymeric substances and organizing into a bilayer structure composed of yeast forms, germ tubes, and early hyphae [56]. hLF1-11 maintains some level of activity during this phase, particularly by interfering with the yeast-to-hyphae transition, which is regulated by the Ras1-cAMP-Efg1 signaling pathway. However, its inhibitory effect becomes weaker at this point, requiring higher concentrations to achieve a comparable response [57]. The synthetic peptides LfcinB17–30 and Lfampin (Lfampin265–284) as well as full-length LF may exert antifungal effects through more complex mechanisms. These peptides are transported into yeast cells via an energy-dependent process, activating mitochondria to synthesize and secrete ATP. The released ATP then interacts with surface receptors, leading to pore formation in the membrane and cell death through a mechanism resembling mitochondrial apoptosis [52]. As the biofilm matures, it forms a dense, three-dimensional structure composed of yeast and filamentous cells embedded in a protective extracellular matrix. This matrix acts as both a physical and biochemical barrier, significantly reducing susceptibility to antifungal agents [56]. At this stage, hLF1-11 exhibits minimal or no antifungal activity even at the highest tested concentration of 176 mg/L, indicating that its efficacy is limited primarily to the early phases of biofilm development [57].

The vaginal environment in healthy women is characterized by an acidic pH, typically ranging from 3.8 to 4.5, which serves as a protective factor against pathogen colonization [58]. Unlike in the phagolysosomal environment of phagocytes, where acidic pH and the presence of reductases promote Fe^2+^ release from LF and ROS generation [15], in the less highly acidic vaginal environment, lactoferrin retains its ability to bind Fe^3+^, providing protection [3,55]. Vaginal acidity (<4.5) also inhibits the transition of *C. albicans* to the hyphal (filamentous) form, thereby limiting its invasive potential and inflammation [3]. In such an environment, the cell wall of *Candida* spp. undergoes pH-dependent remodeling. This process leads to the exposure of immunogenic components, such as β-glucans and chitin, which are masked under neutral pH conditions. Their exposure significantly enhances the fungus’s recognition by immune cells, especially phagocytes, resulting in a stronger host inflammatory response and reducing its ability to evade immune defenses [59]. One of the key antifungal mechanisms of LF is its unique ability to sequester iron in the infection environment [17,18,60]. In vitro study has shown that the iron-free form of lactoferrin significantly reduces the viability of *C. albicans* cells depending on time, temperature, and pH. This effect was most pronounced at neutral pH and 37 °C, with significant fungicidal activity observed after 25 min of exposure to apo-LF. In contrast, this antifungal activity is lost when LF is saturated with iron, underscoring that iron deprivation is a key mechanism limiting the growth of yeast-like fungi. When LF is saturated with iron, its fungicidal properties are abolished, confirming that iron removal from the environment is a crucial factor limiting the growth of yeast-like fungi [60]. Iron deficiency also limits the adhesion of *Candida* spp. cells and the formation of biofilm, a structure that protects the fungus from environmental factors and the immune system [18,55]. Furthermore, apo-LF has effectively reduced biofilm density and its metabolic activity [61].

The host’s immune mechanisms can further support the antimicrobial activity of LF. During phagocytosis, one of the key defenses against *C. albicans* is the production of ROS. Yeast-like fungi have developed survival strategies in the presence of ROS by activating an antioxidant response based on enzymes such as catalase 1, thioredoxin peroxidase, and SOD1 and SOD5, with their expression increasing even before phagocytosis, indicating an early defense reaction. However, once inside the phagocyte, the combination of oxidative and cationic stress (e.g., K^+^ influx into the phagosome) leads to “stress pathway interference”. As a result, the fungus loses the ability to activate protective genes, becoming more susceptible to phagocyte destruction due to inhibiting key transcription factors like Cap1 and kinase Hog1. In response to ROS, the fungus may induce a filamentous form that facilitates escape from phagosomes and survival within the host [62].

LF and other salivary proteins, such as lysozyme, mucins, and defensins, play an essential role as a humoral factor in the oropharyngeal cavity, limiting *Candida* spp. colonization of mucosal surfaces [63]. Its presence supports innate immunity, partly by activating phagocytic mechanisms carried out by neutrophils and macrophages. Although effective phagocytosis usually requires opsonization of *Candida* spp. cells, macrophages can also capture non-opsonized blastoconidia via mannose receptors [63]. Once bound, neutrophils and macrophages eliminate yeast-like fungi by generating ROS and nitric oxide, and this process is regulated by cytokines such as granulocyte–macrophage colony-stimulating factor (GM-CSF), interferons, and prostaglandins [63]. Importantly, LF can act synergistically with other host defense proteins in mucosal secretions, such as lactoperoxidase (LPO), forming the LF–LPO system. Together, they exert a more substantial antimicrobial, including antifungal, effect than either component alone [64]. LF and its peptides support the immune response by inducing the expression of interleukin-1 beta, IL-6, interleukin-12, interleukin-17, transforming growth factor beta, tumor necrosis factor-alpha, as well as enzymes such as inducible nitric oxide synthase and myeloperoxidase. Therefore, exogenous lactoferrin may exert a protective effect in oral candidiasis by reducing inflammation and the number of *Candida* spp. cells on mucosal surfaces [55].

## 5. Lactoferrin in Clinical Practice and the Treatment of Fungal Infections

In the experimental study by Andersson et al. [29], human milk and purified LF were tested against *C. albicans* and *Rhodotorula rubra*, revealing a fungistatic effect. This effect was reversed by the addition of iron, indicating that apo-LF deprives fungi of this essential nutrient. However, in iron-rich environments, LF may even serve as a nutrient source for *Candida* spp. In contrast, Zarzosa-Moreno et al. [52] summarized evidence from multiple studies indicating that apo-LF can exert direct microbicidal effects, including disruption of fungal cell membranes and induction of apoptosis-like processes in pathogenic yeasts, provided that adequate concentrations and conditions are met. These findings emphasize the dual role of LF in fungal infections, as its activity depends heavily on iron saturation status, environmental conditions, and dosage. Species-specific susceptibility of *Candida* spp. strains to LF has been demonstrated in in vitro studies. Among the tested species, *C. tropicalis*, *C. krusei*, and *C. albicans* were more susceptible, whereas *C. glabrata* exhibited the highest resistance. *C. guilliermondii* and *C. parapsilosis* displayed intermediate levels of sensitivity [65]. In the study by Samaranayake et al. [66], which used apo-LF, no significant intragroup differences were observed in the susceptibility of *C. krusei* or *C. albicans* isolates. Differences in susceptibility among *Candida* spp. may be related to variations in cell wall structure and iron acquisition mechanisms, which have been extensively described in the review literature on LF’s activity against various microorganisms [23,52]. Although not yet demonstrated in *Candida* spp., some pathogens are known to utilize holo-LF as an iron source, suggesting a theoretical risk that similar mechanisms could emerge, potentially limiting the therapeutic efficacy of lactoferrin-based strategies [52].

In the study by Giunta et al. [67], administration of 200 mg/day of rhLF to pregnant women led to the normalization of vaginal microbiota and a reduction in IL-6 levels in cervicovaginal fluid, confirming its anti-inflammatory properties and its role in supporting mucosal immunity. In a murine model of vulvovaginal candidiasis, a genetically modified probiotic strain of *Lactobacillus casei* producing bLF demonstrated significant prophylactic and therapeutic effects. Mice treated with this strain showed inhibited hyphal growth of *C. albicans* and a notable reduction in pro-inflammatory cytokines, including IL-17 and interleukin 23 [4]. Additional evidence of the prophylactic efficacy of LF comes from a randomized clinical trial conducted in Italian neonatal intensive care units. In very-low-birth-weight preterm infants, daily oral administration of 100 mg of bLF, alone or in combination with the probiotic *Lactobacillus rhamnosus* GG, significantly reduced the incidence of invasive fungal infections compared to the placebo group. Notably, bLF did not affect the rate of fungal colonization itself but effectively prevented the progression from colonization to systemic infection, highlighting its potential as a natural immunomodulatory agent that enhances the intestinal barrier in neonates [68].

Itraconazole (ITZ), voriconazole (VRZ), and fluconazole (FLC) are azole antifungal agents widely used in the treatment of vaginal infections caused by *Candida* spp., especially in recurrent vulvovaginal candidiasis [69,70,71]. Although they share a common mechanism of action, the efficacy of individual triazoles varies significantly between *Candida* spp., as demonstrated by susceptibility testing in clinical isolates. In cases of azole resistance, alternative topical antifungal agents such as nystatin (NYS), boric acid, flucytosine (5-FC), and amphotericin B (AMB) are occasionally used, primarily in more severe or treatment-resistant infections [69]. In pregnant women, therapeutic recommendations are significantly modified. Due to the potential teratogenicity, oral azoles are not recommended during pregnancy, especially in the first trimester. Their use has been associated with an increased risk of congenital malformations, including skeletal and cardiac abnormalities, as well as spontaneous miscarriages [70]. Clotrimazole (CTZ) is considered the first-line drug for the treatment of vulvovaginal candidiasis during pregnancy [71]. During lactation, the availability of data regarding antifungal drug safety is limited. However, according to current recommendations, topical azoles such as CTZ and miconazole are regarded as safe. In contrast, systemic antifungals such as oral triazoles should be cautiously approached and considered only when the potential benefits to the mother outweigh the possible risks to the infant. In clinical practice, topical formulations are preferred, as they minimize the risk of drug exposure to the breastfeeding infant [72]. In neonates, particularly preterm infants and those with very low birth weight, invasive *Candida* spp. infections pose a serious clinical threat. In neonates, first-line treatment includes amphotericin B deoxycholate and FLC, with the latter recommended only for patients who have not previously received prophylactic therapy with this drug. 5-FC is primarily used in cases involving the central nervous system due to its good penetration into the cerebrospinal fluid [73]. Caspofungin (CAS), a member of the echinocandin class, is increasingly utilized in neonatal intensive care units as salvage therapy or in cases involving resistant *Candida* spp. strains due to its broad-spectrum antifungal activity and favorable safety profile [73]. According to national pediatric practice, mild oral candidiasis in infants is usually treated with topical antifungal therapy. In Poland, NYS is most commonly administered; if symptoms persist or if the infection does not subside, FLC is administered. In addition, great emphasis is placed on hygiene measures: pacifiers and bottles should be regularly boiled or sterilized, and in breastfed infants, simultaneous local treatment of the mother’s nipples is necessary to prevent re-infection. This comprehensive approach improves the effectiveness of treatment and reduces relapses [12,73]. As a dietary supplement combined with antifungal therapy, LF supports the immune system and limits the overgrowth of opportunistic microorganisms such as *Candida* spp. Lactoferrin-derived peptides represent an auspicious adjunctive approach in antifungal treatment. In a study by Danesi et al. [74], synthetic peptides derived from the N-terminal region of LF demonstrated more potent activity against *C. albicans* than the full-length LF molecule. These peptides exhibited direct fungicidal activity and synergistic effects when combined with other antimicrobial agents such as AMB, GM-CSF, lysozyme, or secretory leukocyte protease inhibitor, resulting in enhanced antifungal efficacy. Structural and biochemical studies on the chimeric peptide LFchimera, composed of fragments from lactoferricin and lactoferrampin, demonstrated that its unique structure, formed by linking both peptides via a lysine residue, significantly enhances its ability to destabilize microbial membranes. LFchimera exhibits more vigorous antibacterial and antimicrobial activity than the individual peptides or their mixture. This enhanced activity is believed to result from increased electrostatic interactions with microbial membranes and a structure rich in α-helical content, which may also contribute to its potential antifungal properties [75]. Table 2 presents data on the synergistic effects of various forms of LF combined with antifungal agents against *Candida* spp. strains. Additionally, it was demonstrated that LF in combination with AMB can inhibit the adhesion of *C. albicans* and *C. glabrata* to abiotic surfaces, thereby suppressing biofilm formation and reducing the metabolic activity of cells within mature biofilms [16].

## 6. Conclusions

**Figure 2:**
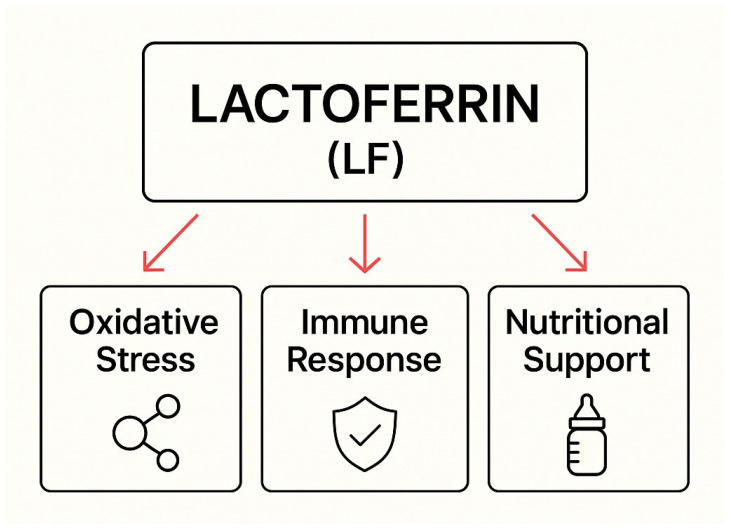
The role of lactoferrin in combating *Candida* spp. infections—a summary of mechanisms of action.

LF exhibits multifaceted activity supporting the body in combating *Candida* spp. infections, which is particularly important in pregnant women and newborns. Iron chelation and the reduction in Fenton reactions decrease oxidative stress, protecting host tissues and supporting phagocytic mechanisms. It modulates the immune response by activating immune cells (neutrophils, macrophages, and T and B lymphocytes) and influencing cytokine production and antigen presentation. LF also acts as a nutritional factor. It delivers bioavailable iron and supports the development of a healthy microbiota, especially in breastfed infants. Its effectiveness in reducing colonization and preventing the progression of infection has been confirmed in clinical studies, and its synergistic action with antifungal drugs makes it a valuable adjunct to therapy. Due to its safety profile, LF holds great potential in preventing and treating fungal infections in high-risk populations. Together, these multifaceted actions illustrate the complexity of lactoferrin’s antifungal mechanisms and highlight its role in host defense strategies, as summarized in Figure 2.

## Figures and Tables

**Table 2:** Clinical and experimental applications of lactoferrin in fungal infections.

Compound	Form and Origin	Target Pathogens	Synergistic Effect	Planktonic and Biofilm Forms	Ref.
LF	Intact bLf (whole protein)	*C. albicans* (including azole-sensitive and -resistant strains)	Synergistic with CTZ; also effective with FLC and ITZ	Planktonic only	[76]
LF-B	Isolated peptide from the N-terminal region of LF	*C. albicans* (including azole-sensitive and -resistant strains)	Synergistic with CTZ	Planktonic only	[76]
LFhyd	Enzymatically digested lactoferrin (mixture of peptides)	*C. albicans* (including azole-sensitive and -resistant strains)	Synergy with azole	Planktonic only	[76]
LF	Dairy-derived bLf (partially digested, low iron saturation)	*C. albicans* and *C. glabrata*	Strong synergy with AMB	Reduces hyphal growth; prevents biofilm formation; less effective against mature biofilm	[16]
hLF1-11	Synthetic peptide from human LF (N-terminal region)	*C. albicans*, *C. glabrata*, *C. tropicalis*, and *C. parapsilosis*	Strong synergy with CAS	Active against planktonic cells; prevents biofilm formation; reduces metabolic activity of mature biofilm (fungistatic)	[61]
LF	Apo-lactoferrin	*C. albicans*, *C. glabrata*, and *C. tropicalis*	Synergistic with FLC; synergistic with 5-FC	Planktonic only	[77]
LF	Native bLf	*C. albicans*, *C. glabrata*, and *C. tropicalis*	Synergistic with FLC	Planktonic only	[77]
hLF(1-11)	Synthetic N-terminal peptide from hLF	*C. albicans* (FLC-sensitive and FLC- resistant), *C. glabrata*, *C. krusei*, *C. tropicalis*, and *C. parapsilosis*	Synergistic with FLC	Planktonic only	[78]

Amphotericin B (AMB); bovine lactoferrin (bLf); caspofungin (CAS); clotrimazole (CTZ); fluconazole (FLC); human lactoferrin (hLf); itraconazole (ITZ); lactoferrin (LF); lactoferrin-derived peptide from bovine LF (LF-B); pepsin hydrolysate of lactoferrin (LFhyd); 5-fluorocytosine (5-FC).

## Abbreviations

The following abbreviations are used in this manuscript:

5-FC	Flucytosine
AMB	Amphotericin B
AMP	Antimicrobial peptide
Apo-LF	Iron-free form of lactoferrin
bCF	Bovine colostrum fortifier
bLf	Bovine lactoferrin
CAS	Caspofungin
CTZ	Clotrimazole
ELFIN trial	Enteral lactoferrin in neonates trial
FLC	Fluconazole
GM-CSF	Granulocyte–macrophage colony-stimulating factor
hLf	Human lactoferrin
Holo-LF	Iron-saturated form of lactoferrin
IL-6	Interleukin 6
ITZ	Itraconazole
LF	Lactoferrin
LOS	Late-onset sepsis
LPO	Lactoperoxidase
NEC	Necrotizing enterocolitis
NYS	Nystatin
rhLF	Recombinant human lactoferrin
ROS	Reactive oxygen species
SOD	Superoxide dismutases
TLR	Toll-like receptors
VRZ	Voriconazole
